# Interactions of Mycotoxin Alternariol with Cytochrome P450 Enzymes and OATP Transporters

**DOI:** 10.3390/metabo13010045

**Published:** 2022-12-28

**Authors:** Eszter Fliszár-Nyúl, Orsolya Ungvári, Ágnes Dombi, Csilla Özvegy-Laczka, Miklós Poór

**Affiliations:** 1 Department of Pharmacology, Faculty of Pharmacy, University of Pécs, Rókus u. 2, H-7624 Pécs, Hungary; 2 Food Biotechnology Research Group, János Szentágothai Research Centre, University of Pécs, Ifjúság útja 20, H-7624 Pécs, Hungary; 3 Drug Resistance Research Group, Institute of Enzymology, Research Centre for Natural Sciences, Eötvös Loránd Research Network, Magyar tudósok krt. 2, H-1117 Budapest, Hungary; 4 Doctoral School of Biology, Institute of Biology, Eötvös Loránd University, Pázmány P. stny. 1/C, H-1117 Budapest, Hungary

**Keywords:** alternariol, CYP enzymes, CYP1A2, OATP transporters, OATP1B1

## Abstract

Alternariol (AOH) is an emerging mycotoxin produced by *Alternaria* strains. The acute toxicity of the mycotoxin is low; however, chronic exposure to AOH may result in the development of endocrine disruptor and/or carcinogenic effects. The toxicokinetic properties of AOH have barely been characterized. Therefore, in this study, we aimed to investigate its interactions with CYP (1A2, 2C9, 2C19, 2D6, and 3A4) enzymes and OATP (1A2, 1B1, 1B3, and 2B1) transporters employing in vitro enzyme assays and OATP overexpressing cells, respectively. Our results demonstrated that AOH is a strong inhibitor of CYP1A2 (IC_50_ = 0.15 μM) and CYP2C9 (IC_50_ = 7.4 μM). Based on the AOH depletion assays in the presence of CYP enzymes, CYP1A2 is mainly involved, while CYP2C19 is moderately involved in the CYP-catalyzed biotransformation of the mycotoxin. AOH proved to be a strong inhibitor of each OATP transporter examined (IC_50_ = 1.9 to 5.4 μM). In addition, both direct and indirect assays suggest the involvement of OATP1B1 in the cellular uptake of the mycotoxin. These findings promote the deeper understanding of certain toxicokinetic interactions of AOH.

## 1. Introduction

Mycotoxins are toxic secondary metabolites of filamentous fungi [1]. *Alternaria* species produce more than 70 mycotoxins [2], including alternariol (AOH), which frequently contaminates berries, tomatoes, oilseeds, grapes, and the corresponding products (e.g., tomato pastes, fruit juices, and wines) [3,4,5]. In previous studies, the genotoxic, immunosuppressive, endocrine disruptor, cytotoxic, and carcinogenic effects of AOH have been reported [4,6,7]. Phase I biotransformation of AOH results in the formation of 2-, 4-, 8-, and 10-hydroxy derivatives [8,9]. Phase II reactions are also involved in the metabolism of AOH: a recent study demonstrated the formation of sulfate conjugates [10], and it is reasonable to hypothesize the production of its glucuronic acid metabolites [9].

Cytochrome P450 (CYP) enzymes contribute to approximately 75% of the phase I biotransformation of xenobiotics [11]. The five human CYP isoforms, which catalyze the majority of these reactions, are CYP1A2, CYP2C9, CYP2C19, CYP2D6, and CYP3A4/5 [11,12]. Since CYP-catalyzed oxidations typically affect the toxicokinetic and toxicodynamic properties of xenobiotics, these enzymes frequently play a major role in the detoxification or toxic activation of certain compounds. The interactions of some mycotoxins (including aflatoxin B1, ochratoxin A, and zearalenone) with CYP enzymes have been reported [13,14,15], while only limited data are available regarding AOH. Based on a previous study, AOH is biotransformed by CYP1A1 and 1A2 enzymes and the mycotoxin can increase the expression of CYP1A1 through the activation of aryl hydrocarbon receptor [16].

Solute carrier organic anion transporting polypeptides (OATPs), present in the cell membrane of epithelial and endothelial cells of the human body, are transporters that mediate the cellular uptake of large (>300 Da), negatively charged, or amphipathic organic molecules [17]. Steroid and thyroid hormones, bile acids, prostaglandins, and bilirubin are typical endogenous substrates of OATPs [18]. Furthermore, multispecific members of the OATP family (OATP1A2, OATP1B1, OATP1B3, and OATP2B1) also recognize various xenobiotics, such as drugs (e.g., statins and certain chemotherapeutics), food components (e.g., flavonoids), and toxins (e.g., α-amanitin and ochratoxin A) [19,20,21]. OATPs 1B1, 1B3, and 2B1 are expressed in human hepatocytes and contribute to the hepatic clearance of their substrates [22]. Furthermore, OATP2B1 and OATP1A2 appear in enterocytes and in the endothelial cells of the blood–brain barrier [23]. These multispecific OATPs are key players in the tissue uptake and clearance of drugs, nutrients, and xenobiotics. Previous studies demonstrated the interaction of the *Aspergillus* mycotoxin ochratoxin A with OATPs [21,24]; however, the effect of AOH on these carriers has not yet been characterized.

In this study, we aimed to investigate the interactions of AOH with CYP (1A2, 2C9, 2C19, 2D6, and 3A4) enzymes and OATP (1A2, 1B1, 1B3, and 2B1) transporters employing in vitro assays with human recombinant enzymes and OATP overexpressing cells, respectively. Our results demonstrated that AOH is a potent inhibitor of CYP1A2, CYP2C9, and the OATPs tested. Furthermore, AOH seems to be a potential substrate for CYP1A2 and OATP1B1. Our findings contribute to the deeper understanding of certain toxicokinetic interactions of AOH.

## 2. Materials and Methods

### 2.1. Reagents

Alternariol was purchased from Cfm Oskar Tropitzsch GmbH (Marketredwitz, Germany). CypExpress human recombinant Cytochrome P450 (1A2, 2C9, 2C19, 2D6, and 3A4) kits, CypExpress Control (CYPnull), testosterone, and 6β-hydroxytestosterone were obtained from Merck (Darmstadt, Germany). CYP1A2 Inhibitor Assay Kit (fluorometric; ab211075) was from Abcam (Cambridge, UK). Diclofenac, 4′-hydroxydiclofenac, S-mephenytoin, 4-hydroxymephenytoin, dextromethorphan, and dextrorphan were purchased from Carbosynth (Berkshire, UK). HPLC grade acetonitrile and methanol were obtained from VWR (Debrecen, Hungary). Nicotinamide adenine dinucleotide phosphate sodium salt and glucose-6-phosphate barium salt were from Reanal (Budapest, Hungary). Stock solution of AOH (40 mM) was prepared in spectroscopic grade dimethyl sulfoxide (DMSO; Fluka, Charlotte, NC, USA) and stored at −20 °C.

### 2.2. Testing the Inhibitory Effect of AOH on CYP450 Enzymes

The potential inhibitory effects of AOH on CYP2C9, 2C19, 2D6, and 3A4 enzymes were tested in vitro employing CypExpress human kits, using the substrates recommended by the U.S. Food and Drug Administration (FDA; https://www.fda.gov/drugs/drug-interactions-labeling/drug-development-and-drug-interactions-table-substrates-inhibitors-and-inducers; accessed on 19 December 2022) (diclofenac, *S*-mephenytoin, dextromethorphan, and testosterone, respectively). The impacts of increasing concentrations of AOH (0.00, 0.05, 0.50, 2.0, 5.0, 10, 25, 30, and 50 μM) were examined on diclofenac 4′-hydroxylation (CYP2C9) [25], *S*-mephenytoin 4-hydroxylation (CYP2C19) [26], dextromethorphan demethylation (CYP2D6) [20], and testosterone 6β-hydroxylation (CYP3A4) [27] as described previously, without modifications. In these assays, the substrates and products were quantified by HPLC-UV [28].

CYP1A2 Inhibitor Assay Kit (Abcam; Cambridge, UK) was applied following the manufacturer’s description, where the inhibitory action of 0.000, 0.001, 0.010, 0.050, 0.10, 0.20, 0.50, 1.25, 3.0, 10, and 25 μM AOH concentrations were tested.

Solvent controls were also applied in each experiment, DMSO concentrations did not exceed 0.13 *v*/*v*%. IC_50_ values were calculated by sigmoidal fitting (Hill1), using the Origin (version 2018, OriginLab Corporation, Northampton, MA, USA) software.

### 2.3. Testing the Biotransformation of AOH by CYP450 Enzymes

To test the potential biotransformation of AOH by CYPs, the changes in the concentrations of AOH were examined in the presence of CYP1A2, 2C9, 2C19, 2D6, or 3A4 enzymes. Samples (final volume: 200 μL) containing AOH (5 μM), glucose-6-phosphate (500 μM), nicotinamide adenine dinucleotide phosphate (NADP^+^, 200 μM), and the enzymes (CypExpress Cytochrome P450 reagents: 15 mg/mL each; including both CYP and glucose-6-phosphate dehydrogenase enzymes) were incubated for 0, 30, 60, 90, and 120 min (700 rpm, 30 °C) in potassium phosphate buffer (0.05 M, pH 7.5). The reaction was stopped by the addition of ice-cold methanol (100 μL), then the samples were centrifuged (10 min, 14,000 g, room temperature). AOH content of the supernatants was directly analyzed by HPLC-FLD (see details in Section 2.7). As a control, CypExpress Control reagent (CYPnull; 15 mg/mL) was also applied, which is a permeabilized and stabilized dried yeast powder (produced using an empty vector) and processed identically to CypExpress Cytochrome P450 products.

### 2.4. Generation and Maintenance of OATP Overexpressing Cell Lines

A431 cell lines overexpressing human OATPs (1A2, 1B1, 1B3, or 2B1) and their mock transfected controls were generated earlier [29,30]. A431 cells expressed no or negligible OATPs compared to the overexpressing cell lines. Cells were cultured in Dulbecco’s modified Eagle medium (DMEM, Thermo Fisher Scientific, Waltham, MA, USA) with 10% fetal bovine serum, 2 mM L-glutamine, 100 U/mL penicillin, and 100 µg/mL streptomycin at 37 °C, with 5% CO_2_. OATP expression was regularly monitored (based on the transport activity of transfected cells), and the cells were used up to 20 passages.

### 2.5. Testing the Inhibitory Effect of AOH on OATP Transporter Function

Interaction between OATPs (1A2, 1B1, 1B3, and 2B1) and AOH was measured in an indirect transport assay [30] with pyranine or sulforhodamine 101 as fluorescent probes [29,31]. A431 cell overexpressing OATPs or their mock transfected controls were seeded on 96-well plates one day prior to the measurements in 200 µL DMEM in 8 × 10^4^ cells/well density. Before the measurement, the medium was removed, and cells were washed three times with 200 µL PBS (phosphate-buffered saline, pH 7.2) at room temperature. Then the cells were preincubated with 50 µL uptake buffer (125 mM NaCl, 4.8 mM KCl, 1.2 mM CaCl_2_, 1.2 mM KH_2_PO_4_, 12 mM MgSO_4_, 25 mM MES, and 5.6 mM glucose, with the pH adjusted using 1 M HEPES and 10 N NaOH, pH 5.5 for OATP1B1/1B3/2B1 and pH 7.4 for OATP1A2) with or without increasing concentrations (0.00, 0.16, 0.31, 0.63, 1.25, 2.5, 5.0, 10, and 20 μM) of AOH for 5 min. The reaction was started when the probe was added in 10 µM (pyranine; OATP1B1), 20 µM (pyranine; OATP2B1 and OATP1B3), or 0.5 µM (sulforhodamine 101; OATP1A2) final concentrations. Incubation times at 37 °C were 10 min (OATP1A2), 15 min (OATP1B1 and OATP2B1), and 30 min (OATP1B3). The reaction was stopped by removing the supernatant and washing the cells three times with ice-cold PBS. Fluorescence was measured in an Enspire plate reader (Perkin Elmer, Waltham, MA, USA) in Ex/Em wavelengths 460/510 nm for pyranine and 586/605 nm for sulforhodamine 101. Bottom reading was applied with two horizontal (X) and two vertical (Y) points, measurement height was 3 mm with 200 flashes. IC_50_ values were calculated by sigmoidal fitting (Hill1), using the Origin (version 2018, OriginLab Corporation, Northampton, MA, USA) software.

### 2.6. Testing the Involvement of OATP1B1 in the Cellular Uptake of AOH

The A431 cell line overexpressing OATP1B1 or mock transfected control were trypsinized, and counted with automated cell counter (TC10, BIORAD, Hercules, CA, USA). The cells were washed with 1 mL buffer (pH 5.5; described above) and centrifuged for 5 min at 1500 g. After the supernatant was removed, 2 × 10^6^ cells in 50 µL volume of the same buffer were handed out into separate Eppendorf tubes, then 50 µL of AOH solutions (diluted in buffer, pH 5.5) was added (final concentrations: 2, 5, or 10 µM). The cells were incubated for 15 min at 37 °C in shaking water bath. The reaction was stopped with 1 mL ice-cold PBS, after which the tubes were centrifuged for 5 min at 1500 g and 4 °C. The supernatant was removed, and the cells were washed with 1 mL ice-cold PBS, then the cell pellet was stored at −80 °C until analyses.

Before HPLC measurements, cell pellets were dissolved in 100 μL of 1 M NaOH solution with vigorous vortexing. After 15 min sonication, the solution was neutralized with 100 μL of 1 M HCl, then 200 μL acetonitrile was added. Samples were vortexed and centrifuged for 10 min at 14,000 g and 4 °C. Thereafter, the supernatant was directly analyzed with HPLC-FLD (see details in Section 2.7).

### 2.7. HPLC Analyses

After CYP450 incubations, the product formation rate was investigated using an integrated HPLC system (Jasco, Tokyo, Japan) built up from an autosampler (AS-4050), a binary pump (PU-4180), and a UV detector (UV-975). Chromatographic data were evaluated employing ChromNAV2 software (Jasco, Tokyo, Japan). Quantitative determinations of 4′-hydroxydiclofenac and diclofenac (CYP2C9), 4-hydroxymephenytoin and mephenytoin (CYP2C19), dextromethorphan and dextrorphan (CYP2D6), as well as testosterone and 6β-hydroxytestosterone (CYP3A4) were carried out as previously reported [28], without modifications.

In CYP experiments, AOH was quantified with the above-described HPLC system, except that a fluorescence detector (FP-920; Jasco, Tokyo, Japan) was applied. Samples (20 μL) were driven through a Phenomenex Security Guard (C18, 4.0 × 3.0 mm) precolumn (Phenomenex, Torrance, CA, USA) linked to a Kinetex EVO-C18 (250 × 4.6 mm, 5 μm; Phenomenex) analytical column with 1.0 mL/min flow rate, at room temperature. For the isocratic elution, a mobile phase with 1 mM phosphoric acid (pH 3.0) and acetonitrile (65:35 *v*/*v*%) was applied. AOH was detected at 455 nm (λ_ex_ = 345 nm). Limit of detection (LOD) and limit of quantification (LOQ) were established as the lowest concentrations when the signal-to-noise ratios were three and ten, respectively. The major validation parameters of this HPLC-FLD method were the following: linearity (R^2^ = 0.997, in the 100–1000 nM concentration range); LOD = 100 nM; LOQ = 200 nM; intraday precision = 2.9% (n = 7); accuracy = 3.4% (n = 7).

To examine the OATP1B1-mediated uptake of AOH in A431 cells, our recently reported magnesium-sensitized HPLC-FLD method was applied [32]. Briefly, samples (20 μL) were driven through a Security Guard (C8, 4.0 × 3.0 mm; Phenomenex, Torrance, CA, USA) precolumn linked to a Kinetex C8 (100 × 4.6 mm, 5 μm; Phenomenex, Torrance, CA, USA) analytical column. The isocratic elution was performed with 10 mM HEPES buffer (pH 7.0) and acetonitrile (70:30 *v*/*v*%) containing 50 mM MgCl_2_, using 1.0 mL/min flow rate at room temperature. AOH was quantified using 345 nm and 455 nm excitation and emission wavelengths, respectively. The major validation parameters of this HPLC-FLD method were the following: linearity (R^2^ = 0.999 in the 20–1000 nM concentration range); LOD = 10 nM; LOQ = 20 nM; intraday precision = 2.2% (n = 7); accuracy = 2.6% (n = 7).

### 2.8. Testing AOH-OATP Interaction in Competitive Counterflow and Efflux Assays

The assays were performed as described recently [33]. Cells were seeded on 96-well plates one day prior to the measurement. After washing three times with PBS at room temperature, cells were incubated for 15 min with 8-acetoxy-1,3,6-trisulfopyrene (Ace, 5 µM). After this “preloading” stage, the supernatant was removed, and the cells were incubated with the same amount of the probe (Counterflow assay) or without the probe in buffer pH 5.5 (Efflux assay) in the absence and the presence of AOH for further 20 min. Fixed concentrations of AOH were selected based on the IC_50_ values determined in the transport inhibition assay. Estrone-3-sulfate (E1S, 50 μM) and formaldehyde (PFA, 0.5%) served as reference substrate and inhibitor, respectively. Cells were washed three times with ice-cold PBS, then NaOH solution (0.1 N, 200 µL/well) was added. After 20 min, fluorescence was measured with an Enspire plate reader in Ex/Em wavelengths 460/510 nm. Bottom reading was applied with two horizontal (X) and two vertical (Y) points, measurement height was 3 mm with 200 flashes. Fluorescence was compared to the preloaded control incubated with Ace alone, considered as 100%.

### 2.9. Data Analyses

Data display mean and standard error of the mean (±SEM) values, derived from at least three independent experiments. Statistical significance (*p* < 0.05 and *p* < 0.01) was evaluated based on one-way ANOVA test followed by Tukey’s post hoc test (version 21, IBM SPSS Statistics, Armonk, NY, USA).

## 3. Results and Discussion

### 3.1. Interaction of AOH with CYP Enzymes

**Figure 1:**
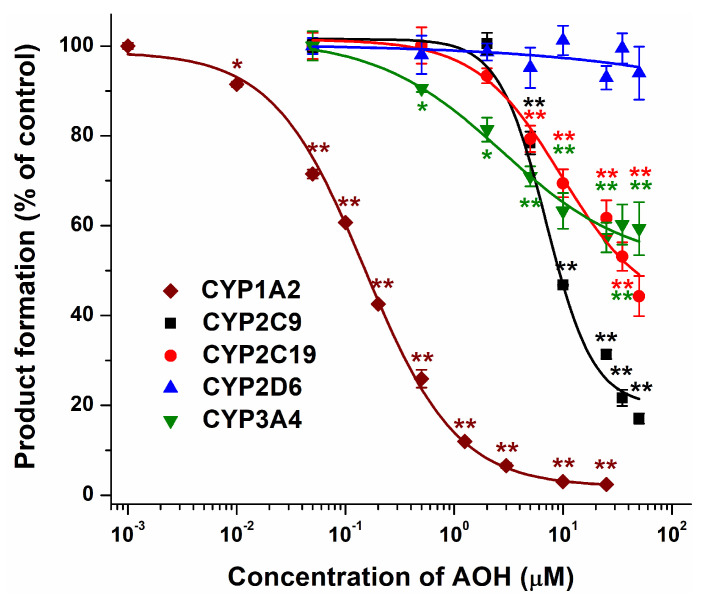
Concentration-dependent inhibitory effects of AOH (0–50 μM) on CYP1A2, CYP2C9, CYP2C19, CYP2D6, and CYP3A4 enzymes (n = 3; * *p* < 0.05, ** *p* < 0.01).

The interactions of AOH with CYP enzymes were investigated employing 0 to 50 μM mycotoxin concentrations. AOH induced the concentration-dependent inhibition of the CYP enzymes tested, except CYP2D6-catalyzed dextromethorphan demethylation, which was not affected even by 50 μM AOH (Figure 1). It is not surprising because typically amines, which can be protonated under physiological circumstances, can interact with CYP2D6 [34]. Among the CYP enzymes examined, AOH proved to be the most potent inhibitor of CYP1A2 (IC_50_ = 0.15 μM), causing statistically significant (*p* < 0.01) and close to complete inhibitions at 0.05 μM and 10 μM concentrations, respectively. Furthermore, the mycotoxin strongly inhibited the CYP2C9 enzyme (IC_50_ = 7.4 μM), leading to more than an 80% decrease in the product formation at 50 μM concentration. At 5 μM concentration, AOH significantly (*p* < 0.01) inhibited CYP2C19; nevertheless, the highest AOH concentration applied (50 μM) caused only a 55% decrease in 4-hydroxymephenytoin production (Figure 1). Finally, AOH inhibited CYP3A4 enzyme; however, the mycotoxin did not cause a further relevant decrease in the metabolite formation above 10 μM concentration (approximately 40% inhibition) (Figure 1).

Urolithins are colon metabolites of ellagitannins [35]; they have a very similar chemical structure to *Alternaria* mycotoxins. In agreement with our results, a previous study demonstrated the inhibitory effects of urolithins (e.g., urolithin A, B, and C) on CYP1A2 and CYP1B1 enzymes [36].

The inhibitory actions of certain other mycotoxins on CYP enzymes have also been reported. Zearalenone showed a strong inhibitory effect on CYP2C9-catalyzed tolbutamide hydroxylation (IC_50_ = 0.54 μM), while it was only a weak inhibitor of CYP2D6-mediated dextromethorphan *O*-demethylation (IC_50_ = 55.4 μM) [37]. T-2 toxin and zearalenone markedly reduced CYP3A4-catalyzed midazolam hydroxylation in vitro (IC_50_ values were 27.0 and 1.1 μM, respectively); in addition, zearalenone increased the oral bioavailability of midazolam in pigs [37].

**Figure 2:**
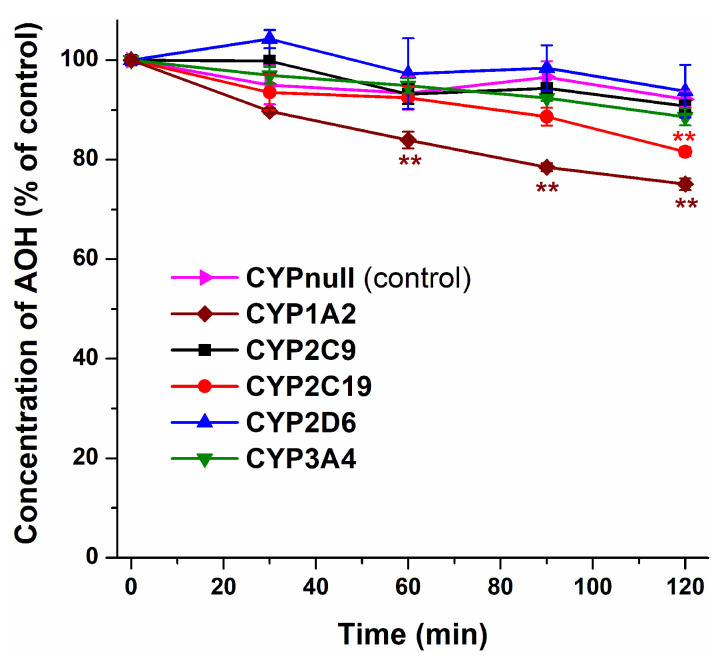
Time-dependent changes in the concentrations of AOH (5 μM) in the presence of the different CYP isoenzymes (see experimental details in 2.3; n = 3; ** *p* < 0.01). Statistical evaluation was performed compared to the incubation with the same amount of CYPnull (15 mg/mL), which is a permeabilized and stabilized dried yeast powder (produced using an empty vector) and processed identically to CypExpress Cytochrome P450 products.

To test the potential biotransformation of the mycotoxin by CYP enzymes, AOH was incubated with CYP1A2, 2C9, 2C19, 2D6, and 3A4. Even after a 120 min incubation, our results demonstrated no relevant changes in AOH levels in the presence of CYP2C9, CYP2D6, and CYP3A4. These incubates showed very similar data to the control (CYPnull) (Figure 2). CYP2C19 caused only a slight decrease in AOH levels, showing a statistically significant difference (*p* < 0.01) only after a 120 min incubation. However, CYP1A2 enzyme induced a concentration-dependent decrease in AOH concentrations, leading to a 16% and 25% depletion of AOH after 60 min and 120 min incubations, respectively (Figure 2).

Based on our current knowledge, this is the first study to examine the inhibitory action of AOH on CYP enzymes. Considering the above-listed data, AOH is a weak inhibitor of CYP2C19 and CYP3A4, while it exerts very strong and moderately strong inhibition on CYP1A2 and CYP2C9 enzymes, respectively (Figure 1). Furthermore, based on these observations, we did not see the relevant involvement of CYP2C9, CYP2D6, and CYP3A4 in the metabolism of AOH. On the other hand, CYP2C19 and mainly CYP1A2 seem to be important CYP enzymes regarding the biotransformation of this mycotoxin (Figure 2). In an earlier study, the CYP-catalyzed biotransformation of AOH was examined using pooled human hepatic microsomes, suggesting the CYP1A-mediated metabolism of AOH with the minor involvement of CYP2C19 and CYP3A4 [38]. Our study confirms the importance of the CYP1A2 and CYP2C19 enzymes.

### 3.2. Interaction of AOH with OATP Transporters

**Figure 3:**
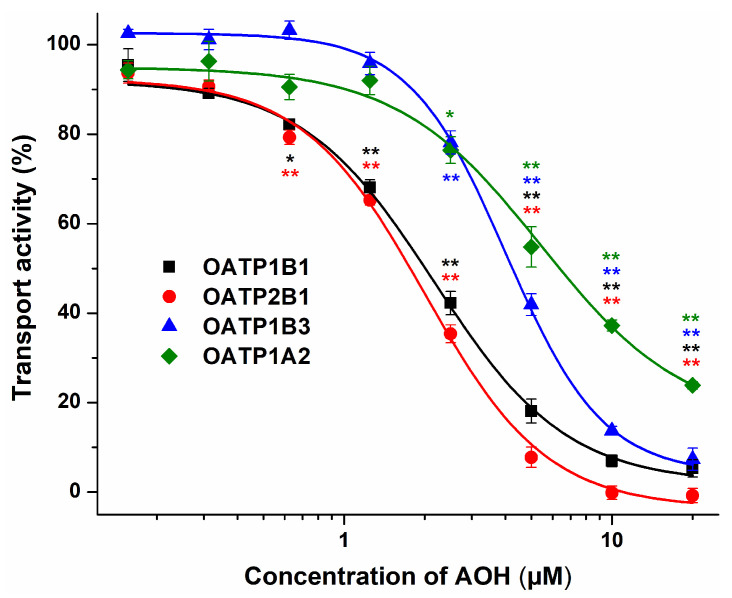
Inhibition of dye uptake in the presence of increasing AOH concentrations (0–20 μM) in A431 cells overexpressing human OATPs (see experimental details in Section 2.5; n = 3; * *p* < 0.05, ** *p* < 0.01).

First, the potential inhibitory impact of AOH on OATP transporters was examined, employing pyranine or sulforhodamine 101, previously documented as fluorescent test substrates [29,31]. As demonstrated in Figure 3, AOH proved to be a potent inhibitor of each OATP tested, showing the strongest inhibitory actions on OATP2B1 and OATP1B1 (IC_50_ values were 1.9 µM and 2.0 µM, respectively), while slightly lower effects were observed regarding OATP1B3 and OATP1A2 (IC_50_ values were 4.1 µM and 5.4 µM, respectively).

**Figure 4:**
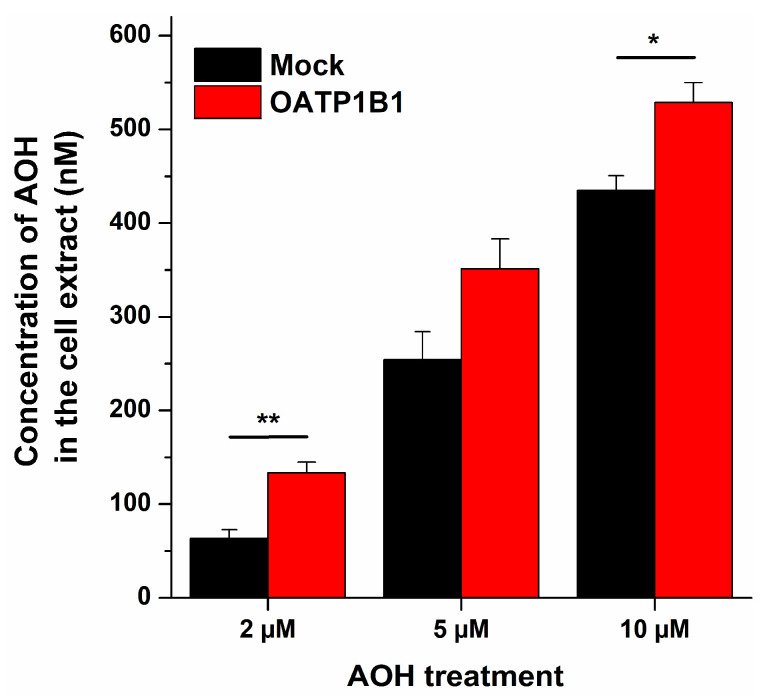
AOH concentrations in A431 cell extracts after 15 min incubation with 2, 5, or 10 μM mycotoxin solutions. AOH was quantified by HPLC-FLD (see experimental details in Section 2.6 and Section 2.7; n = 6; * *p* < 0.05, ** *p* < 0.01).

To determine whether AOH is a potential transported substrate of OATP1B1, AOH uptake was investigated in OATP expressing and mock transfected A431 cells. In these experiments, cells were incubated with increasing concentrations of AOH, then the cellular mycotoxin concentration was quantified by HPLC-FLD. OATP1B1 expressing cells showed higher cellular AOH levels compared to mock cells (Figure 4), suggesting the OATP1B1-mediated transport of the mycotoxin. However, likely due to the rapid and significant membrane association of the mycotoxin, we also found relatively high levels of AOH in the mock cells. Therefore, to confirm the involvement of OATP1B1 in the cellular uptake of AOH, further experiments were performed.

**Figure 5:**
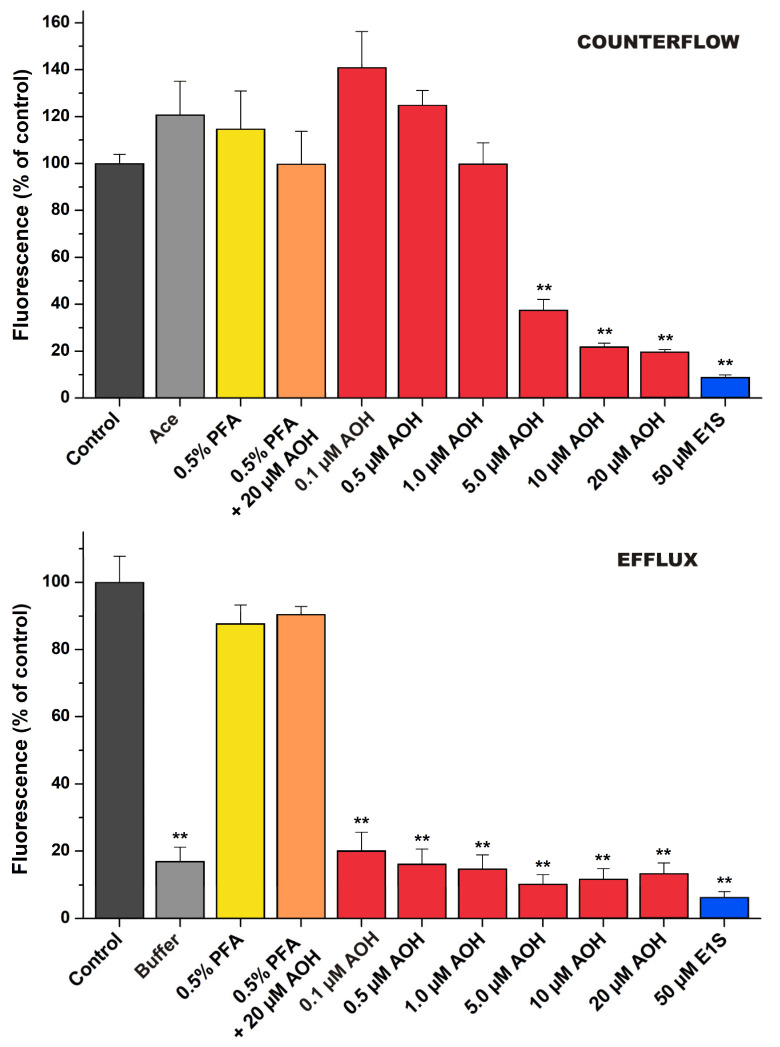
Effects of AOH on dye transport in CCF (**top**) and efflux (**bottom**) assays (see experimental details in Section 2.8; n = 3; ** *p* < 0.01).

Recently, a novel method, termed as competitive counterflow (CCF), was established that can distinguish transported substrates and non-transported inhibitors of OATP2B1 or OATP1A2 [39,40]. The method is based on the exchanger function of OATPs, by which even large organic substrates can be swapped between the opposite sites of the cell membrane. Lately, using the fluorogenic substrate 8-acetoxy-1,3,6-trisulfopyrene (Ace), we developed the CCF for OATP1B1 [33]. The method comprises two steps. First, the cells are loaded with Ace to a steady state (by the function of OATP1B1). Next, the investigated compound (AOH in the current study) is added. The addition of a substrate (E1S used here as a reference substrate) at high enough amounts (ten-fold of its IC_50_ value) triggers the efflux of the probe, leading to a decrease in the fluorescence signal in the CCF (Figure 5, top) and not inhibiting efflux (Figure 5, bottom). On the other hand, non-transported inhibitors (we used formaldehyde, a chemical crosslinker here) blocks the efflux of the probe (Figure 5, bottom) and there is no change in the fluorescence in the CCF. Figure 5 indicates that AOH added in excess (already at two-fold of its IC_50_) generated a decrease in the fluorescence signal in the CCF, while not inhibiting efflux. These observations indicate that AOH is a potential substrate of OATP1B1.

Only limited information is available regarding the interactions of mycotoxins with OATP transporters. Nevertheless, based on previous studies, certain OATPs may have significant involvement in the tissue uptake and accumulation of mycotoxins ochratoxin A [21,24] and deoxynivalenol [41]. In our study, AOH showed strong, concentration-dependent inhibitory action on each OATP transporter tested (IC_50_ = 1.9 to 5.4 μM). Therefore, the potential involvement of OATP1B1 in the cellular uptake of the mycotoxin was also examined. We measured higher concentrations of AOH in OATP1B1 expressing vs. the mock cells (Figure 4). In addition, counterflow and efflux assays were performed, where we could distinguish non-transported inhibitors and transported substrates of OATP1B1 [33]. In these experiments, AOH behaved similarly to the reference substrate E1S (Figure 5). These observations strongly suggest that AOH is not only an inhibitor but also a substrate of the OATP1B1 transporter.

## 4. Conclusions

In summary, CYP enzymes and OATP transporters are involved in the pharmacokinetics and/or toxicokinetics of several drugs and xenobiotics. Since toxicokinetic interactions of mycotoxin AOH have barely been characterized, in the current study, we aimed to investigate its interactions with CYP (1A2, 2C9, 2C19, 2D6, and 3A4) enzymes and OATP (1A2, 1B1, 1B3, and 2B1) transporters. Considering the low oral bioavailability of AOH as well as its typically nanomolar concentrations in the circulation [4,9,10], it seems to be unlikely that AOH can considerably interfere with the CYP-catalyzed biotransformation and/or the OATP-mediated transport of drugs. However, AOH concentration may be significantly higher in the intestinal tract than in the circulation, suggesting the possible inhibition of OATP2B1-mediated absorption of certain compounds. In addition, the involvement of OATPs in the tissue uptake of mycotoxin AOH may have toxicological importance. Considering the endocrine disruptor effects of AOH [4,7], further studies are reasonable to test the potential role of OATP transporters in the uptake of the mycotoxins into the target organs.

## Data Availability

Data is not publicly available due to privacy or ethical restrictions.
